# The effect of age on the relationship between cardiac and vascular function

**DOI:** 10.1016/j.mad.2015.11.001

**Published:** 2016-01

**Authors:** David Houghton, Thomas W. Jones, Sophie Cassidy, Mario Siervo, Guy A. MacGowan, Michael I. Trenell, Djordje G. Jakovljevic

**Affiliations:** a Institute of Cellular Medicine, MoveLab, Medical School, Newcastle University, Newcastle upon Tyne, UK; b Institute of Neurosciences and Newcastle University Institute for Ageing, Newcastle University, Newcastle upon Tyne, UK; c Human Nutrition Research Centre, Institute of Cellular Medicine, Newcastle University, Campus for Ageing and Vitality, Newcastle upon Tyne, UK; d Department of Cardiology, Freeman Hospital, Newcastle upon Tyne, UK; e Institute of Genetic Medicine, Newcastle University, Newcastle upon Tyne, UK; f RCUK Centre for Ageing and Vitality, Newcastle University, Newcastle upon Tyne, UK

**Keywords:** Ageing, Cardiac function, Vascular function, Exercise test

## Abstract

Age-related changes in cardiac and vascular function are associated with increased risk of cardiovascular mortality and morbidity. The aim of the present study was to define the effect of age on the relationship between cardiac and vascular function. Haemodynamic and gas exchange measurements were performed at rest and peak exercise in healthy individuals. Augmentation index was measured at rest. Cardiac power output, a measure of overall cardiac function, was calculated as the product of cardiac output and mean arterial blood pressure. Augmentation index was significantly higher in older than younger participants (27.7 ± 10.1 vs. 2.5 ± 10.1%, *P* < 0.01). Older people demonstrated significantly higher stroke volume and mean arterial blood pressure (*P* < 0.05), but lower heart rate (145 ± 13 vs. 172 ± 10 beats/min, *P* < 0.01) and peak oxygen consumption (22.5 ± 5.2 vs. 41.2 ± 8.4 ml/kg/min, *P* < 0.01). There was a significant negative relationship between augmentation index and peak exercise cardiac power output (*r* = −0.73, *P* = 0.02) and cardiac output (*r* = −0.69, *P* = 0.03) in older participants. Older people maintain maximal cardiac function due to increased stroke volume. Vascular function is a strong predictor of overall cardiac function in older but in not younger people.

## 1 Introduction

Age-associated changes in cardiac and vascular function are identified as a major risk factor for cardiovascular morbidity and mortality, with older patients having a higher risk of having cardiovascular morbidity and mortality (Westerhof and O’Rourke, 1995, Shih et al., 2011, McEniery et al., 2005, Lakatta, 2002, Franklin, 2005). The age associated changes that occur are present even in the absence of hypertension or clinically apparent cardiovascular disease (Lakatta, 2002). Age-associated cardiovascular changes are the key determinant of the decline in functional capacity in older age (Astrand, 1960, Ogawa et al., 1992). Stiffening of arteries is commonly reported and leads to an increased systolic blood pressure (Takazawa et al., 1996, Jakovljevic et al., 2010). To overcome these vascular changes and increased afterload, the heart needs to impart more energy into the vascular system by generating more pressure. This may lead to an increase in left ventricular wall thickness and mass with ageing, lowering the threshold for clinical signs and symptoms. (Lakatta, 2002). Earlier identification of cardiovascular disease (CVD) may lead to improved prognosis and quality of life in older people. Assessment of vascular function is one commonly used method recognised as an important prognostic index and a potential target for therapeutic intervention in CVD and impact upon clinical care (DeLoach and Townsend, 2008, Weber et al., 2004).

A non-invasive assessment of pulse wave reflection i.e. pulse wave analysis and calculation of augmentation index can be used to determine vascular function and provide information regarding arterial compliance. Central arterial compliance, as measured by augmentation index informs arterial stiffness calculations and overall vascular function (Patvardhan et al., 2011). Augmentation index has been identified as an independent CVD risk factor and is strongly correlated with atherosclerosis (Weber et al., 2004). Previous studies have demonstrated a strong relationship between central aortic and radial vascular compliance (Patvardhan et al., 2011, Munir et al., 2008), supporting the use of radial tonometry in vascular function assessment, with a number of studies confirming its reliability (McEniery et al., 2005, Pauca et al., 2001, Fantin et al., 2007, Kohara et al., 2005, Wilkinson et al., 1998, O’Rourke et al., 1999, Takazawa et al., 1996).

Currently, there is limited information on how vascular function impacts on overall cardiac function in older subjects at rest and during peak exercise. This can be demonstrated with assessment of the relationship between augmentation index as a measure of vascular function and cardiac power output, a unique index of cardiac pumping capability and overall cardiac function that integrates both the pressure and flow-generating capacities of the heart (Jakovljevic et al., 2010, Tan, 1986). Considering the strong interaction between vascular and cardiac ageing, we hypothesize that augmentation index can be used to predict maximal cardiac pumping capability which has been shown to be the strongest predictor of mortality in patients with heart failure (Williams et al., 2001). We also aim to evaluate differences in vascular function and cardiac response to exercise between young and older people.

## 2 Materials and methods

Thirty healthy individuals i.e. 20 young (10 women), aged between 20 and 30 years, and 10 older (6 women) aged between 60 and 71 years participated in the study. All participants performed <60 min of moderate to vigorous activity per week (defined as 3–6 and >6 metabolic equivalent of task, MET), respectively, as defined by the Centers for Disease Control (CDC) and Prevention and the American College of Sports Medicine (ACSM), were non-smokers, normotensive, free from any cardiac and respiratory disorders and no medication 3 months prior to study commencement, as determined during screening and consent. All procedures were according to Declaration of Helsinki and the study was approved by the local research ethics committee. All participants gave their written informed consent. Subjects were instructed to abstain from eating for a >2 h before each test and from vigorous exercise 24 h prior. Subjects were also instructed not to consume alcohol or caffeine containing foods and beverages on test days. All tests were conducting between 9 and 10 am to ensure assessments were consistent and minimise effects of day to day activities. Upon arrival at the laboratory participants were asked to lay in a supine position for 10 min. Blood pressure was measured in duplicate in the brachial-artery of participant’s non dominant arm. Mean arterial pressure was calculated as the diastolic pressure plus one third of the pulse pressure.

### 2.1 Augmentation index measurement

Pulse wave analysis was used to assess vascular function by measuring augmentation index using the SphygmoCor (AtCor Medical, NSW, Australia) under resting condition. As per the manufactures instructions a high fidelity micro-manometer was used to apply pressure and therefore flatten the radial artery in the non-dominant hand at the wrist. The probe was then placed over the vessel at the point where a strong arterial pulse can be ascertained. The probe was pressed down against the artery, ensuring that the artery was not occluded and a signal was produced on the portable computer. Data was collected for a minimum of 20 sequential waveforms into a portable computer with amplitude in excess of 500 mV with accuracy for waveforms of over 85% to ensure validity of measurements. The data were then analysed using the integral software to generate the mean peripheral and corresponding waveform. Augmentation index was then calculated automatically using the SphygmoCor software. Augmentation index was calculated as the difference between the first systolic peak and the second systolic peak of the central arterial waveform, which was expressed as a percentage of pulse pressure.

### 2.2 Progressive exercise test and cardiac output measurement

Following assessment of arterial stiffness all participants performed maximal graded cardiopulmonary exercise test on an electro-magnetically braked semi-recumbent cycle ergometer (Corival, Lode, Groningen, Netherlands) with simultaneous measurements of respiratory and gas exchange (Cortex metalyser 3B, Leipzig, Germany) and non-invasive cardiac output data using bioreactance method (NICOM^®^, Cheetah Medical, Deleware, USA), previously detailed (Jakovljevic et al., 2012, Jakovljevic et al., 2015, Jones et al., 2015). Briefly, bioreactance estimates cardiac output from analysis of the frequency of relative phase shifts of electrical current applied across the thorax using four dual-surface electrodes. Signals were applied to and recorded from the left and right sides of the thorax; these signals are processed separately and averaged after digital processing. The signal processing unit of the system determines the relative phase shift between the input signal relative to the output signal. The phase shift occurs due to instantaneous changes in blood flow in the aorta. Cardiac output is subsequently estimated as the product of stroke volume and heart rate.

Once participants were connected to the measurement units, 5 min of resting data were recorded. Participants than continued to cycle using graded exercise stress test protocol until volitional exhaustion was reached or were not able to maintain a cadence of 60–70 revolutions per minute. Blood pressure was determined automatically at rest, during and at peak exercise using an automated blood pressure system (SunTech Tango, SunTech Medical, Inc., Morrisville, USA). The graded exercise test was considered maximal if participants achieved any two of the following criteria: no change in oxygen consumption despite further increase in workload, (i) a respiratory exchange ratio of 1.15 or greater, or (ii) ≥90% age predicted maximum heart rate (Winter et al., 2006). Maximal oxygen consumption and cardiac output were defined as the mean values obtained over the last minute of exercise. Anaerobic threshold was automatically calculated using v-slope method (Tan, 1986). Cardiac pumping capability was represented by cardiac power output (expressed in watts) and calculated from the product of cardiac output and mean arterial pressure, as previously described (Tan, 1986, Jones et al., 2015, Goldspink et al., 2009, Fleg et al., 2005). Systemic vascular resistance to blood flow was estimated as the ratio between mean arterial pressure and cardiac output and multiplied by a factor of 80 to convert units to dynes per second per centimeter to the fifth power. Arteriovenous oxygen difference was calculated as the ratio between oxygen consumption and cardiac output.

### 2.3 Statistical analyses

Statistical analyses were performed using SPSS statistical analysis software (Version 19, IBM, USA). Normality of distribution was assessed using a Kolmogorov–Smirnov test. Relationship between arterial stiffness (i.e. augmentation index) and cardiac pumping capability (i.e. cardiac power output) was assessed using Pearson’s product moment coefficient of correlation. Independent sample *t*-tests were used to identify differences between metabolic, gas exchange and haemodynamic data between young and older study participants. Statistical significance was indicated if *P* ≤ 0.05. Data are presented as mean ± SD unless otherwise indicated.

## 3 Results

Physical characteristics of the young participants were: age 26.3 (±4.2) years, height 171.5 (±8.0) cm, body mass 67.4 (±7.9) kg, and peak oxygen consumption 41.5 (±8.7) ml/kg/min. The characteristics for the older participants were: age 67.0 (±2.8) years, height 165.7 (±9.1) cm, body mass 74.3 (±7.9) kg and peak oxygen consumption 23.4 (±5.1) ml/kg/min. There were no significant differences in height and body mass between the young and older participants (*P* > 0.05). The resting and peak exercise data for metabolic, gas exchange and haemodynamic variables are presented in Table 1, Table 2, respectively. Oxygen consumption, carbon dioxide output, stroke volume, cardiac output, systemic vascular resistance to blood flow, augmentation index were significantly different between the young and older participants (*P* < 0.05), as were metabolic and gas exchange variables at peak exercise (*P* < 0.05). Central haemodynamic measures including stroke volume, heart rate and mean arterial blood pressure were significantly different between the young and older participants (*P *< 0.05). Cardiac power output, measured at rest and peak exercise, was not significantly different between the young and older people.

### 3.1 Relationship between augmentation index and cardiac and metabolic function at peak exercise

**Fig. 1:**
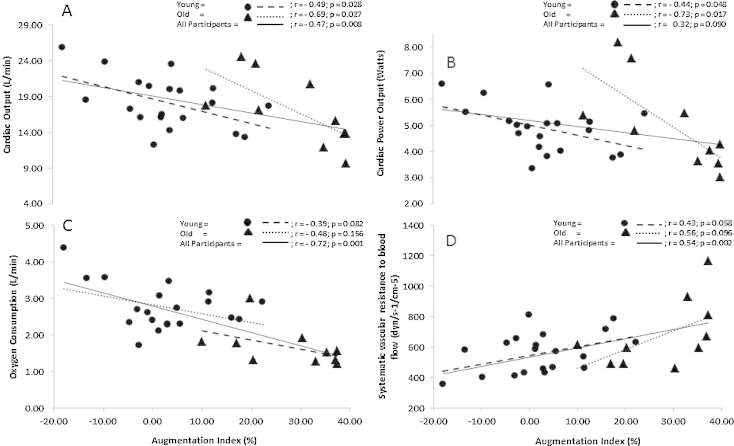
Relationship between augmentation index and (A) peak exercise cardiac output, (B) peak exercise cardiac power output, (C) peak exercise oxygen consumption and (D) systemic vascular resistance to blood flow (Values are means) (*n* = 30).

There was a strong negative correlation between augmentation index and cardiac output (*r* = −0.69, *P* = 0.027) and cardiac power output in older participants (*r *= −0.73, *P* = 0.017), whereas in younger participants these relationships were only moderate (Fig. 1A and B). When data from all participants are considered together, augmentation index was significantly correlated with peak exercise oxygen consumption (*r* = −0.72, *P* = 0.001, Fig. 1C) and systemic vascular resistance (*r* = 0.54, *P* = 0.002, Fig. 1D). There was a moderate positive correlation between augmentation index and peak exercise systolic blood pressure (*r* = 0.38, *P* = 0.014) when data from all participants were combined. However, subgroup analysis according to age revealed no significant differences between augmentation index, peak oxygen consumption and systematic vascular resistance (*P* > 0.05). Additional analysis also revealed no significant relationship between augmentation index and height (*P* > 0.05).

Data for all participants revealed strong negative correlations between augmentation index and peak exercise carbon dioxide output (*r* = −0.67, *P* = 0.001), minute ventilation (*r* = −0.70, *P* = 0.001), and heart rate (*r* = −0.61, *P* = 0.001). There was also a moderate negative correlation between augmentation index and skeletal muscles’ ability to extract oxygen i.e. arteriovenous oxygen difference (r = −0.49, *P* = 0.005) and anaerobic threshold (*r* = 0.52, *P* = 0.003) when all participants data were analysed. However, the relationships reported here were not present when subgroup analysis based on age were performed (*P* > 0.05).

### 3.2 Relationship between augmentation index, cardiac and metabolic function at rest

When all participants data were combined during resting conditions, there was a moderate negative correlation between augmentation index stoke volume (*r* = −0.44, *P* = 0.015) and cardiac output (*r* = −0.43, *P* = 0.017) and moderate positive correlation with systemic vascular resistance (*r* = 0.53, *P* = 0.002). Subgroup analysis according to age revealed no significant correlations between augmentation index and any of the haemodynamic variables assessed including systolic blood pressure (*P* > 0.05).

There was a strong negative correlation between augmentation index and oxygen consumption in older participants only (*r* = −0.71, *P* = 0.022). Augmentation index was not significantly correlated with other metabolic and gas exchange measurements at rest including carbon dioxide output, oxygen extraction, respiratory exchange ratio and ventilation (*P* > 0.05).

## 4 Discussion

The present study is the first to demonstrate the strength of the relationship between vascular function and cardiac pumping capability. Additionally, the current study confirms the effect of ageing on cardiac and metabolic response to a maximal graded exercise test. Primary findings indicate a significant relationship between augmentation index and peak exercise cardiac power output, implying that vascular function is a strong predictor of cardiac pumping capability in older people. The results also reveal that vascular function significantly declines with ageing as demonstrated by an increase in augmentation index. This is despite the lack of significant difference between the young and older participants in maximal cardiac pumping capability (i.e. cardiac power output). The increased peak exercise stroke volume in the older participants contributed to preserved cardiac output despite a significant decline in maximal heart rate with age.

It has been previously suggested that cardiovascular function and functional capacity decline with ageing (Lakatta, 2002, Jakovljevic et al., 2015, Lakatta and Levy, 2003). The data in the present study indicate that older people have an increased augmentation index, systemic vascular resistance and systolic blood pressure, which is suggestive of a decrease in vascular function and increased arterial stiffness. The older participants also had reduced cardiac output and stroke volume under resting conditions, which is consistent with previous investigations (Ogawa et al., 1992, Beere et al., 1999, Kaess et al., 2012). It should, however, be noted that augmentation index was only moderately correlated with resting cardiac output and stroke volume, but not with cardiac power output. These data combined suggest that vascular function has a limited effect upon cardiac function. This is an interesting finding because, to some extent, it contradicts previous suggestions that the age-related decline in arterial function directly influences the decline in cardiac function (Lakatta, 2002, Lakatta and Levy, 2003). The data presented here demonstrate a significant age-related decline in both (i) arterial function with increased augmentation index, and (ii) cardiac function e.g. reduced cardiac output and stroke volume at rest in older people. However, due to a the lack of a strong relationship between augmentation index and cardiac output and/or cardiac power output, it is reasonable to suggest that age-related decline in vascular function does not necessary lead to reduced cardiac function at rest.

The age-related differences in cardiovascular function are apparent when the heart is exposed to physiological stress such as exercise testing (Lakatta and Levy, 2003, Stratton et al., 1994). The impact of age on cardiac response to maximal stress testing has been debated for a long time. While several investigators have reported a significant reduction in maximal cardiac output and cardiac power output in older individuals (Goldspink et al., 2009, Stratton et al., 1994, Tanaka et al., 2000), others including the present study reported no significant changes (Lakatta and Levy, 2003, Fleg et al., 1994). A plausible explanation for the maintenance of cardiac output at peak exercise with ageing is an increased stroke volume as demonstrated in the present and in previous studies (Lakatta and Levy, 2003, Fleg et al., 1994). This ability to increase and/or maintain stroke volume at peak exercise is a result of the age associated cardiac changes in end diastolic volume through the Frank Starling mechanism (Lakatta and Levy, 2003, Fleg et al., 1995). In the present study the older group demonstrated a 40% increase in stroke volume from rest to peak exercise. In contrast, there was no significant difference between resting and peak exercise stroke volume in younger participants. This increase in stoke volume in the older group reflects the ability of the ageing heart to maintain cardiac output despite a reduction in heart rate which has been previously reported (Stratton et al., 1994, Fleg et al., 1994).

The strong relationship between augmentation index and peak exercise cardiac power output found in the present study clearly suggests that vascular function as measured through augmentation index can be used as an independent predictor of maximal cardiac pumping capability in older, but not younger people. This is an important finding that can have clinical applications. For example, in older people and those with heart failure, who are unable to undertake exercise stress test with haemodynamic monitoring, augmentation index may be used as a surrogate measure of overall function and pumping capability of the heart i.e. cardiac power output—the strongest predictor of mortality in heart failure (Williams et al., 2001).

Furthermore, our findings are consistent with those of previous studies demonstrating a significant increase in augmentation index, suggesting an age-related decrease in vascular function and an increase in arterial stiffness (McEniery et al., 2005, Patvardhan et al., 2011, Fantin et al., 2007, Kohara et al., 2005, Roman et al., 2007, Arnett et al., 1994). Values for augmentation index found in the present study are similar to those previously reported (McEniery et al., 2005, Ogawa et al., 1992, Winter et al., 2006). As arterial function declines the arteries stiffen, resulting in a premature return of the reflected wave during systolic contraction. This then leads to a rise systolic blood pressure (Laurent et al., 2006) and the development of isolated systolic blood pressure (Kaess et al., 2012). Similar findings were reported at peak exercise by Beere et al. (1999), who also found that those with a higher systemic vascular resistance demonstrated a higher systolic blood pressure. These data substantiate the findings of the current study suggesting that increased systemic vascular resistance and systolic blood pressure at peak exercise are related to a reduced arterial function and increased arterial stiffness. This places increased stress on the heart’s ability to pump the blood and can lead to left ventricular hypertrophy due to a pressure overload. Interestingly, current data show that under resting condition the augmentation index was not significantly related to systolic or diastolic blood pressure regardless of age. This observation was also previously reported (Kohara et al., 2005) further suggesting that age-related increase in arterial blood pressure may be contributed to factors other than reduced arterial function and arterial stiffness.

Lastly, the data in the present study demonstrate a significant reduction in oxygen consumption at rest and peak exercise in the older group. A reduction in oxygen uptake due to ageing can be attributed to an attenuated cardiac output, and to some extent arteriovenous oxygen difference (Beere et al., 1999). Stratton et al. (1994) demonstrated that a reduction in maximal oxygen uptake in older people was associated with an increased systolic blood pressure and reduced arterial compliance. Similar results were also reported by Ogawa et al. (1992) who demonstrated a 40% reduction in maximal oxygen uptake in the older group which was associated with reduced cardiac output and arteriovenous oxygen difference. Combined with a reduced vascular function and increased systematic vascular function, these data indicate that skeletal muscles demonstrate a diminished capacity to extract oxygen in older people, particularly during exercise, as demonstrated in the present by a reduction in arteriovenous oxygen difference in the older group. Our data also suggest that reduced oxygen consumption at peak exercise in older people is mainly due to a reduced ability of skeletal muscles to extract oxygen rather than the heart’s ability to deliver it. These suggestions are concurrent with previous studies that have demonstrated an age related decline in muscle mass, strength and endurance (Peterson et al., 2012). This reduction in muscle mass is associated with a decline in the number and function of the mitochondria present in skeletal muscle in the elderly (Menshikova et al., 2006), and ultimately an inability to extract oxygen. Furthermore a strong relationship between augmentation index and resting and maximal oxygen consumption found in the present study suggest that vascular function may play a significant role in the reduced resting and exercise metabolic functions associated with ageing (Fleg et al., 2005, Fleg et al., 1994, Fleg et al., 1995).

In the present study the following limitations should be considered. Firstly, the measurement of hemodynamic variables was obtained non-invasively, which is not recognised as the ‘gold standard’. Although the validity of bioreactance has previously been reported, future work looking at a comparison between the NICOM and invasive ‘gold standard’ methods for measuring hemodynamic variables is warranted. Secondly the measurement of augmentation index was made at the radial artery and not the carotid artery. It is possible that values obtained at the radial artery are different from those obtained at the carotid artery due to the pressure contour changes as it travels from the central aorta to the peripheral arteries. Although measurement of the carotid artery may provide a more accurate assessment of central pulse pressure, this can be difficult because the surrounding area to the carotid artery is loose tissue making it difficult to obtain consistent applantation and reliable results (Wilkinson et al., 1998). Thirdly, the sample size was relatively small, particularly within the older group, limiting generalization of data. Nonetheless, the numbers included were sufficient to report for the first time the strength of the relationship between vascular and cardiac function. Finally, subgroup analysis according to gender was not possible due to the small sample size.

In conclusion, the findings of the present study indicate a strong relationship between augmentation index and peak exercise cardiac power output, suggesting that a measure of vascular function can be used as a predictor of cardiac pumping capability in older people. The data further show a significant decline in vascular but not cardiac function with ageing. Future studies are warranted to investigate the relationship between arterial stiffness and cardiac power output in older people with heart failure where both vascular and cardiac function are significantly reduced and strongly associated with poor short- and long-term prognosis.

## 5 Clinical perspective

The data demonstrate a strong relationship between augmentation index, a measure of vascular function, and peak exercise cardiac power output, a direct measure of cardiac pumping capability and overall cardiac function. Augmentation index therefore can be used as a surrogate measure of cardiac pumping capability in older people. This finding can be of a particular importance in clinical settings where direct assessment of cardiac function during stress testing is strongly suggested to assess prognosis and enhance risk stratification (i.e. in heart failure) but may not be feasible due to technical demands and patients’ ability.

## Author contributions

Study conceived and designed by DGJ. Data collection performed by DH, TWJ, SC, MS and DGJ. Data extraction and analyses performed by DH and TWJ. Interpretation of data and preparation of manuscript performed by DGJ, DH, TWJ, SC, MS, GAM and MIT.

## Conflict of interest

This study is not industry sponsored; DH, TWJ, SC, GAM, MS, MIT and DGJ report no conflict of interests.

## Disclosures

This work was supported by the UK National Institute for Health Research Biomedical Research Centre for Ageing and Age-related Diseases award to Newcastle upon Tyne Hospitals NHS Foundation Trust. MIT is supported by the UK National Institute for Health Research Senior Research Fellowship. DGJ is supported by the Research Councils UK Centre for Ageing and Vitality. The funding sources did not have a direct role in the design, collection, analysis or interpretation of data, nor in the manuscript preparation, which is solely the remit of the author(s).

## Figures and Tables

**Table 1:** Metabolic and gas exchange variables at rest and peak exercise.

	Rest				Peak exercise			
	Young	Old	*P* value	All participants	Young	Old	*P* value	All participants
Oxygen consumption (ml/kg/min)	4.9 ± 0.8	3.7 ± 0.4	0.001	4.5 ± 0.9	41.2 ± 8.4	22.5 ± 5.2	0.001	35.0 ± 11.6
Carbon dioxide output (L/min)	0.3 ± 0.1	0.2 ± 0.04	0.040	0.3 ± 0.6	3.9 ± 1.2	1.9 ± 1.2	0.001	3.2 ± 1.0
Oxygen consumption (L/min)	0.4 ± 0.1	0.3 ± 0.1	0.002	0.3 ± 0.1	2.8 ± 0.6	1.7 ± 0.5	0.001	2.4 ± 0.6
Respiratory exchange ratio	0.9 ± 0.1	0.9 ± 0.04	0.241	0.9 ± 0.1	1.2 ± 0.1	1.1 ± 0.04	0.103	1.2 ± 0.1
Minute ventilation (L/min)	10.2 ± 2.4	9.2 ± 2.0	0.207	9.9 ± 1.5	91.7 ± 18.9	59.0 ± 18.3	0.001	80.8 ± 18.9
Arteriovenous oxygen difference (ml O_2_/100 ml of blood)	5.4 ± 1.7	4.3 ± 1.6	0.083	5.0 ± 1.9	15.4 ± 2.6	10.1 ± 1.8	0.001	13.6 ± 3.7
Anaerobic threshold (ml/kg/min)	–	–	–	–	25.3 ± 8.2	13.6 ± 2.2	0.001	21.4 ± 9.2

**Table 2:** Haemodynamic variables at rest and peak exercise.

	Rest				Peak exercise			
	Young	Old	*P* value	All participants	Young	Old	*P* value	All participants
Stroke volume (ml/beat)	107.6 ± 19.8	82.3 ± 19.3	0.003	99.2 ± 22.8	106.0 ± 22.4	135.2 ± 43.9	0.020	115.7 ± 31.5
Heart rate (beats/min)	65.0 ± 8.0	71.9 ± 9.6	0.111	67.9 ± 8.3	171.9 ± 10.1	145.1 ± 13.3	0.001	163.0 ± 16.6
Cardiac output (L/min)	7.1 ± 1.2	5.8 ± 1.0	0.004	6.6 ± 1.4	18.2 ± 3.7	16.8 ± 4.9	0.462	17.7 ± 2.0
Cardiac power output (Watts)	1.5 ± 0.3	1.3 ± 0.3	0.082	1.4 ± 0.3	4.9 ± 0.9	5.0 ± 1.7	0.855	4.9 ± 0.6
Systolic blood pressure (mmHg)	132 ± 14	138 ± 17	0.241	133 ± 14	196 ± 19	208 ± 16	0.080	200 ± 17
Diastolic blood pressure (mmHg	78 ± 8	81 ± 9	0.325	79 ± 8	84 ± 12	95 ± 15	0.074	88 ± 15
Mean arterial pressure (mmHg)	95 ± 7	100 ± 10	0.205	97 ± 8	122 ± 9	133 ± 12	0.024	125 ± 9
Systemic vascular resistance to blood flow (dyn/s^−1^/cm^−5^)	1117 ± 211	1427 ± 279	0.008	1220 ± 247	560 ± 132	682 ± 224	0.137	601 ± 75
Augmentation index (%)	2.5 ± 10.1	27.7 ± 10.1	0.001	10.9 ± 15.4				
